# The Effect of Rubber–Metal Interactions on the Mechanical, Magneto–Mechanical, and Electrical Properties of Iron, Aluminum, and Hybrid Filler-Based Styrene–Butadiene Rubber Composites

**DOI:** 10.3390/polym16172424

**Published:** 2024-08-27

**Authors:** Md Najib Alam, Vineet Kumar, Seok-U Jeong, Sang-Shin Park

**Affiliations:** 1 School of Mechanical Engineering, Yeungnam University, 280, Daehak-ro, Gyeongsan 38541, Republic of Korea; mdnajib.alam3@gmail.com (M.N.A.); vineetfri@gmail.com (V.K.); 2 Graduate School of Mechanical Engineering, Yeungnam University, 280, Daehak-ro, Gyeongsan 38541, Republic of Korea; fffu123@naver.com

**Keywords:** styrene–butadiene rubber, metallic fillers, conducting rubber composites, magneto–mechanical properties, swelling properties

## Abstract

Multifunctional stretchable rubber composites are gaining attention due to their unique electrical, mechanical, and magnetic properties. However, their high production costs pose economic challenges. This study explores the use of cost-effective metal powders—iron, aluminum, and their 1:1 (vol/vol) hybrid filler—in styrene–butadiene rubber composites, varying from 10 to 20 vol%. The effects of these metal particles on the mechanical, electrical, morphological, and swelling properties were investigated. Metal particles generally act as non-reinforcing fillers but can significantly enhance the mechanical modulus, electrical, and magnetic properties based on the filler structure and the filler–rubber interactions. Iron-based composites exhibit significant electrical conductivity and excellent magnetic properties. Aluminum enhances the modulus, while the combination yields average mechanical properties with added magnetic characteristics. Iron demonstrates higher reactivity with sulfur-based crosslinking ingredients, adversely affecting the rubber matrix’s crosslinks, as shown by swelling tests. This reactivity is attributed to iron’s transition metal characteristics. At 20 vol%, iron-filled composites display the highest magnetic anisotropic effect on toughness (~25%) under a magnetic field by permanent magnets and excellent electrical conductivity (1.5 × 10^−2^ S/m). While iron significantly boosts the electrical and magnetic properties, higher filler amounts degrade the mechanical properties. These composites are currently suitable for electrical and smart mechanical applications, but incorporating reinforcing fillers could enhance their robustness for broader applications.

## 1. Introduction

Rubber composites are increasingly being utilized in various industrial and engineering applications [1,2]. Traditionally, fillers like silica and clay have been used for mechanical reinforcement, but incorporating fillers with superior electrical, thermal, and magnetic properties can enhance the performance of rubber composites in these areas [3,4,5]. Rubber inherently has poor thermal and electrical conductivity, but these can be significantly improved by adding functional fillers, expanding the applications to electrical, mechanical, and thermal management systems [6,7]. For instance, rubber composites with iron powder fillers exhibit excellent magnetic properties, making them suitable for advanced technologies such as sensors, actuators, and magnetorheological dampers [8,9,10]. Additionally, rubber composites with metal fillers offer microwave absorption properties, making them highly effective for radiation shielding [11]. Metal-based conductive rubber composites are valuable for discharging static electricity in triboelectric systems, which is crucial for safety in environments with flammable gases [12]. Furthermore, due to their stretchability, rubber–metal composites can serve as stretchable conductors in various applications [13,14,15].

Different metals, such as aluminum, nickel, cobalt, iron, and zinc, are commonly used to make rubber–metal composites. Vinod et al. [16] studied the effects of heat, ozone, and high-energy radiation on aluminum powder-filled natural rubber composites, finding that these composites exhibit better resistance in mechanical properties under such conditions. In another investigation, Vinod et al. [17] found that aluminum improves the thermal conductivity of nitrile rubber composites. Anuar et al. [18] examined the curing, mechanical, and electrical properties of aluminum and zinc-filled natural rubber composites. They observed that zinc-based composites have higher tensile strength and elongation properties but a lower modulus compared to aluminum-based composites. They also found that electrical resistance is influenced by the size of the filler materials, with aluminum-filled rubber composites showing lower electrical resistance due to the larger particle size compared to zinc-based composites. Nassar et al. [19] investigated the effect of aluminum content up to 60 wt% in styrene–butadiene rubber composites, finding enhanced dielectric constant and dielectric loss values. Due to their magnetic properties and electrical conductivities, iron, cobalt, and nickel are useful for fabricating conducting rubber composites with tunable mechanical properties in various magnetic fields, making them ideal for smart magnetorheological elastomeric applications [20]. Zainudin et al. [8] found that cobalt, as a magnetic filler in silicone rubber, improved the magnetorheological properties and enhanced the electrical conductivity, making it useful as a sensor composite. Metal powders with a low aspect ratio in the filler structure demonstrate excellent electrical conductivity in rubber composites. Due to their low aspect ratio, even small deformations lead to significant changes in electrical resistivity in the composite, allowing for extremely high piezoresistive strain sensitivity [15,21]. Various studies [22,23,24,25] indicate that rubber composites with magnetic metals can improve microwave absorption and electromagnetic interference shielding efficiencies, along with enhancing electrical and magnetic losses. Additionally, iron filler-based rubber composites can be used as stretchable electrical induction heating materials for various technological applications [26].

Previous studies [16,17,18,19,20,21,22,23,24,25,26] have primarily focused on the thermal, electrical, and mechanical properties of metal-based polymer composites. However, the different chemical characteristics of various metals can lead to distinct effects on the physical and chemical properties of rubber composites. For instance, metallic iron, such as carbonyl iron particles, exhibits strong physical interactions with benzene π-electrons and d-electron systems in SBR rubber composites, thereby improving the mechanical properties [27]. Besides these physical interactions, there may also be chemical interactions with rubber additives. Most rubbers require curing with additives such as sulfur and accelerators, which can chemically interact with metals and influence the final chemical crosslinks. Both physical interactions and chemical crosslinks between rubber chains significantly impact the ultimate mechanical properties of rubber composites. Therefore, it is essential to evaluate all possible interactions of metals in rubber composites to fully understand their effects on mechanical and electrical properties. However, no studies have yet explored physical and chemical interactions separately. By considering these interactions independently, we can gain a clearer understanding of the electrical, mechanical, and other properties of metal-based rubber composites.

Initially, three metal powders—iron, copper, and aluminum—were selected as fillers for styrene–butadiene rubber-based composites. However, after curing the rubber composite with copper powder, an unconventional sticky compound was formed. This could be due to the high reactivity of copper with the sulfur-based curing ingredients, which significantly affects the crosslinking formation. Consequently, further experiments with copper powder were not conducted.

In this study, two classes of metal elements based on their periodic properties—iron (d-block) and aluminum (p-block)—were investigated as fillers in a styrene–butadiene rubber system. Iron is known for its strong magnetic properties, while aluminum exhibits excellent thermal conductivity, making it suitable for magnetic applications requiring enhanced thermal management. Styrene–butadiene rubber was selected as the matrix due to its stretchability, high modulus, and effective physical interactions with various fillers, which are advantageous for applications in high-load-bearing mechanical systems. Comparative analyses were conducted to examine the influence of these different metals on the physical and chemical properties of the resulting rubber composites. Swelling studies were performed to assess the chemical effects of the metals on crosslink formations within the rubber matrix. Additionally, considering its electrical conductivity and magnetic properties, iron was evaluated for its magneto-mechanical characteristics, aiming at potential applications in conductive magnetorheological elastomers.

## 2. Materials and Methods

### 2.1. Materials

**Figure 1:**
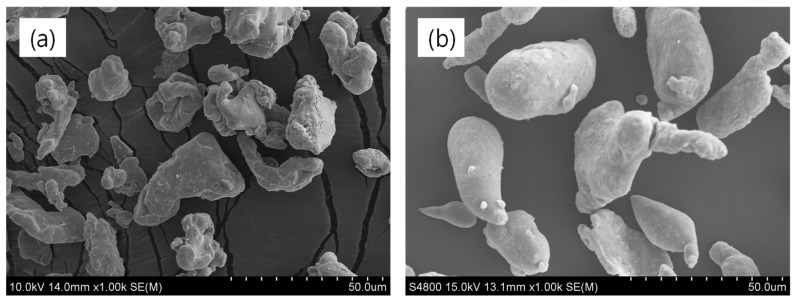
SEM images; (**a**) iron particles and (**b**) aluminum particles.

Styrene–butadiene rubber (SBR 1502, ~23.5% styrene content) was procured from Kumho Petrochemicals, Republic of Korea. A masterbatch rubber was prepared using the following curing ingredients in parts per hundred grams of rubber (phr): zinc oxide (5 phr), stearic acid (2 phr), accelerator N-tert-butyl-benzothiazole sulfonamide (1.75 phr), accelerator tetramethyl thiuram disulfide (1 phr), and sulfur (1.5 phr). The mixing process was carried out on an open two-roll mill. Electrolytic iron powder with an average particle size of 400 mesh was sourced from AO Metal Corporation Ltd., Republic of Korea. Aluminum powder, with an average particle size of 325 mesh, and the solvent toluene were obtained from Daejung Chemicals & Metals Co. Ltd., Siheung, Republic of Korea. The scanning electron micrographs of the two metals are shown in Figure 1a,b. It is evident from these figures that iron particles (Figure 1a) are relatively smaller in size with more uneven structures compared to the aluminum particles (Figure 1b).

### 2.2. Fabrication of Rubber Composites

A specific amount of masterbatch rubber, as indicated in Table 1, was soaked in 100 mL of toluene for 24 h in a glass jar. The soaked rubber was then homogenized through mechanical stirring, resulting in a highly viscous slurry. In a separate container, varying amounts (0–20 vol%) of iron or aluminum powder, or their 1:1 (*v*/*v*) hybrid, were mixed with 20 mL of toluene and subjected to 30 min of sonication. This filler solution was subsequently added to the homogenized rubber slurry and mixed vigorously for about 10 min using mechanical stirring. The filler-mixed rubber slurry was then placed in an oven at 80 °C for 24 h to allow for solvent evaporation. To determine the volume percentage of filler in the rubber composites, the weight/density formula was applied, using theoretical density values of 7.87 g/cm^3^ for iron and 2.7 g/cm^3^ for aluminum. The volume fraction of rubber was calculated based on the measured density of unfilled rubber, which was 0.94 g/cm^3^. The different mixing compositions and filler amounts are detailed in Table 1.

**Figure 2:**
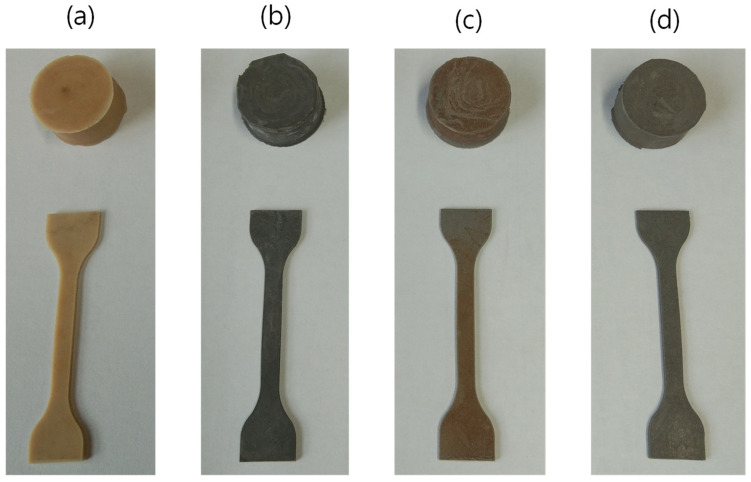
Camera images; (**a**) SBR-unfilled, (**b**) SBR/20vol% Fe, (**c**) SBR/20vol% Al, and (**d**) SBR/20vol% Hyb.

After drying, the compounded rubber was vulcanized at 150 °C in a hot press molding machine into cylindrical or sheet-like shapes, as described in previous studies [27,28]. The colors and physical shapes of the unfilled and highest filler-loaded vulcanizates are illustrated in Figure 2a–d for both the unfilled and highest filler-loaded compounds. The vulcanized rubber samples were then stored in a refrigerator to control environmental effects. They were removed from the refrigerator one day before measuring their physical and mechanical properties.

### 2.3. Measurements of the Mechanical Properties

Tensile, compressive, and stress relaxation tests were conducted to investigate the mechanical properties of the rubber composites using a universal testing machine (Lloyd UTM, Westminster, UK) equipped with a 1 kN load cell. For the tensile test, dumbbell-shaped test pieces were cut from the vulcanized sheet according to the ISO-37 [29], type-2 standard. The test was performed with a constant motor speed of 300 mm/min and a gauge length of 25 mm. For the compressive test, cylindrical samples with a diameter of 20 mm and a height of 10 mm were used. The motor speed was set at 2 mm/min, and the ultimate compressive strain was 30%. To investigate the stress relaxation behavior, the cylindrical sample was compressed to 30%, and the load relaxation was observed for up to 60 s.

### 2.4. Magneto-Mechanical Properties

The magneto-mechanical properties in the presence of neodymium permanent magnets (Grade: N35 NdFeB, Remanence ~1.2 T, dimensions: d = 30 mm and h = 10 mm) were studied similarly to the compressive mechanical properties. The compressive mechanical characteristics exhibited changes due to the magnetic field-induced anisotropic effect in the ferromagnetic rubber composites. The anisotropic effects on various mechanical properties were determined by comparing the mechanical properties in the presence and absence of the magnetic field.

### 2.5. Electrical Properties

The electrical properties, including the resistivity and conductivity, of the rubber composites were measured using cylindrical samples. The two-probe resistance measurement method was employed, with two copper electrodes placed on opposite sides of the cylinder. A digital multimeter (Keysight, 34461A, Santa Rosa, CA, USA) was used for these measurements. The multimeter’s maximum resistance measurement limit was 100 megaohms.

### 2.6. Filler Dispersion

Field-emission scanning electron microscopy (FE-SEM, S-4800, Hitachi, Tokyo, Japan) was used to study the dispersion of the fillers in the rubber matrix. Prior to capturing the SEM images, the tensile fractured surfaces were coated with gold using a sputtering machine.

### 2.7. Swelling Studies and Crosslink Density Measurements

Rubber can swell in a solvent until it reaches thermodynamic equilibrium. It is known that as the chemical crosslinking density increases, the rubber becomes less swellable. Therefore, information about the chemical crosslinks achieved in the rubber composites in the presence of metallic fillers can be obtained. It is assumed that due to the strong metallic bonds in the metallic filler systems, there will be no solvent swelling of the fillers. Consequently, any observed solvent swelling must be attributed to the rubber phase, and the crosslink density in the rubber phase can be determined experimentally.

To study the swelling characteristics of the rubber compounds, cylindrical test pieces were immersed in toluene (solvent) for 7 days to reach a swelling equilibrium. From the swelling equilibrium data, two swelling index values were determined: one for the total composite and one for the fraction of rubber in the composite. The swelling of the solvent relative to the fraction of rubber was used to determine the crosslink density in the rubber matrix.

The swelling index values were calculated using the formula shown in Equation (1).

(1) $$Swelling\;Index=\frac{Swelled\;volume\;of\;solvent}{Volume\;of\;whole\;composite\;or\;volume\;of\;rubber\;fraction}$$

The crosslink density in the rubber matrix was determined using the Flory–Rehner equation [30], as depicted in Equations (2) and (3).

(2) $$V_{c}=\frac{-\{ln(1-V_{r})+V_{r}+\chi V_{r}^{2}\}}{V_{s}d_{r}\left(V_{r}^{\frac{1}{3}}-\frac{V_{r}}{2}\right)}$$

In Equation (2), V_r_ represents the volume fraction of rubber in the swollen phase, and V_c_ denotes the crosslink density. The interaction parameter χ = 0.49 applies to the SBR/toluene (solvent) system, while V_s_ = 106.2 signifies the molar volume of toluene (solvent), and d_r_ = 0.94 g/cm^3^ stands for the density of rubber.

(3) $$V_{r}=\left(\frac{\frac{w_{r}}{d_{r}}}{\frac{w_{r}}{d_{r}}+\frac{w_{s}}{d_{s}}}\right)$$

In Equation (3), w_r_ and w_s_ denote the weights, and d_r_ and d_s_ represent the densities of the rubber and solvent, respectively. The density of toluene was assumed to be 0.87 g/cm^3^.

## 3. Results and Discussion

### 3.1. Mechanical Properties

**Figure 3:**
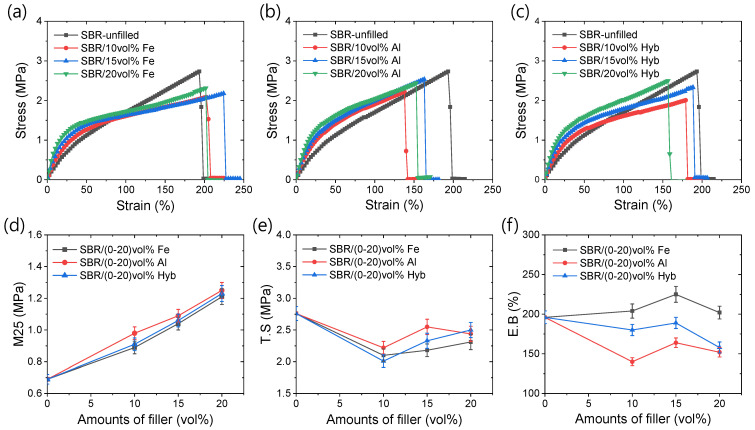
Tensile mechanical properties; (**a**) stress–strain of iron-based composites, (**b**) stress–strain of aluminum-based composites, (**c**) stress–strain of hybrid filler-based composites, (**d**) M25, (**e**) T.S, and (**f**) E.B.

Figure 3a–c illustrates the tensile stress–strain curves for different rubber composites. At lower strains, the stress values increase with higher filler amounts, indicating improved mechanical strength. However, at higher strains, the stress decreases relative to the unfilled rubber, possibly due to diminished filler–polymer interactions, given that the fillers are micrometer-sized particles [27]. Notably, in Figure 3a, iron-based composites exhibit comparable or slightly higher toughness than unfilled rubber, suggesting distinct physical interactions with iron particles [27]. In contrast, such interactions appear absent in aluminum-based composites as shown in Figure 3b.

Figure 3d displays the modulus at 25% strain (M25) values for various composites as a function of filler amounts. It is evident that the modulus increases with higher filler concentrations, with aluminum-based composites exhibiting higher modulus values compared to iron and hybrid filler-based composites. However, these differences diminish as the filler content increases. Fröhlich et al. [31] propose that the modulus of filled rubber depends on the filler–filler interactions, in-rubber structures, hydrodynamic effects, and the rubber network. Given that both iron and aluminum particles exceed micrometer sizes, their filler–filler interactions and in-rubber structures are expected to contribute less to the modulus values at lower filler contents. Instead, hydrodynamic effects likely play a major role due to the rigidity and size of the filler particles. Moreover, the rubber network significantly influences the modulus, as indicated by the stress–strain properties and modulus of the unfilled rubber. Aluminum particles, being larger, are likely to enhance the modulus values more through hydrodynamic effects. Additionally, aluminum’s lower chemical influence on crosslink formation compared to iron, noted in swelling investigations, suggests another reason for their modulus enhancement. As filler content increases, the filler–filler interactions and in-rubber structures develop. Iron particles, more irregular and capable of strong interactions with SBR’s benzene ring, may form more robust in-rubber structures at higher filler levels [27,32,33]. This is reflected in Figure 3d by similar modulus values at higher filler contents and the pronounced effects observed in compressive properties, where fillers aggregate rather than separate as seen in the tensile testing.

Figure 3e displays the tensile strength (T.S) values of various rubber composites. The figure indicates that the T.S initially decreases with 10 vol% filler content in the composites, followed by an increase with higher filler amounts. These findings suggest the formation of a potentially stronger filler network at an optimal filler concentration, likely due to enhanced filler–polymer interactions. However, the T.S value of the rubber composites does not match that of the unfilled rubber, possibly attributable to the micron size of the particles.

The maximum stretchability of rubber composites is influenced by factors such as the homogeneity of the filler distribution, the filler–rubber interactions, and the rigidity of the rubber networks. Figure 3f plots the elongation at break (E.B) values of the rubber composites. From this figure, it is evident that aluminum filler-based composites exhibit lower stretchability, whereas the iron-based composites demonstrate higher stretchability. This trend aligns with the observations of the tensile strength values, suggesting that more stretchable filler networks may form due to uniform filler dispersion and improved filler–rubber interactions up to an optimal filler amount. Further insights from swelling properties, discussed later, indicate that iron-based rubber composites, characterized by lower crosslink densities, enable higher stretchability compared to other filler systems.

**Figure 4:**
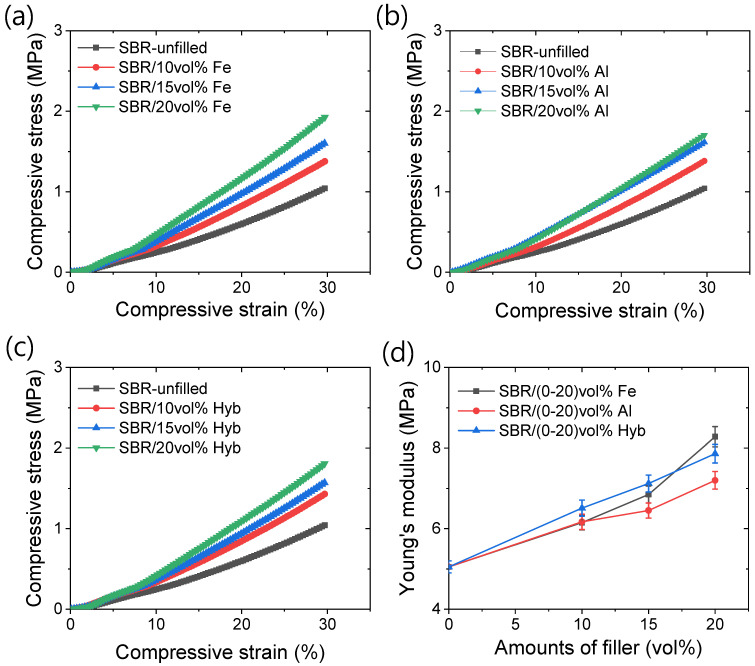
Compressive mechanical properties; (**a**) stress–strain of iron-based composites, (**b**) stress–strain of aluminum-based composites, (**c**) stress–strain of hybrid filler-based composites, and (**d**) Young’s modulus.

Figure 4a–d presents various compressive mechanical properties. From the compressive stress–strain curves shown in Figure 4a–c, it is evident that the slope of the compressive stress–strain increases more rapidly with compression for the iron-based composites compared to the aluminum and hybrid filler-based composites. It is important to note that during compression, the filler particles come closer together unlike in the tensile deformation where they tend to separate. The irregular structure of iron particles allows them to potentially form in-rubber structures after reaching a certain filler content, effectively occluding the rubber within the filler matrix [31]. This shielding prevents rubber deformation, leading to an increase in the effective filler content and contributing to the compressive modulus. In Figure 4d, the Young’s modulus values show that aluminum-based composites have a lower modulus compared to iron-based composites. At lower filler contents, hybrid filler-based composites exhibit better modulus values, possibly due to stronger polymer networks and the presence of iron particles that promote in-rubber structures. At the highest filler content, a significant number of in-rubber structures may form due to strong filler–polymer interactions with iron particles [27,31], resulting in the highest Young’s modulus.

**Figure 5:**
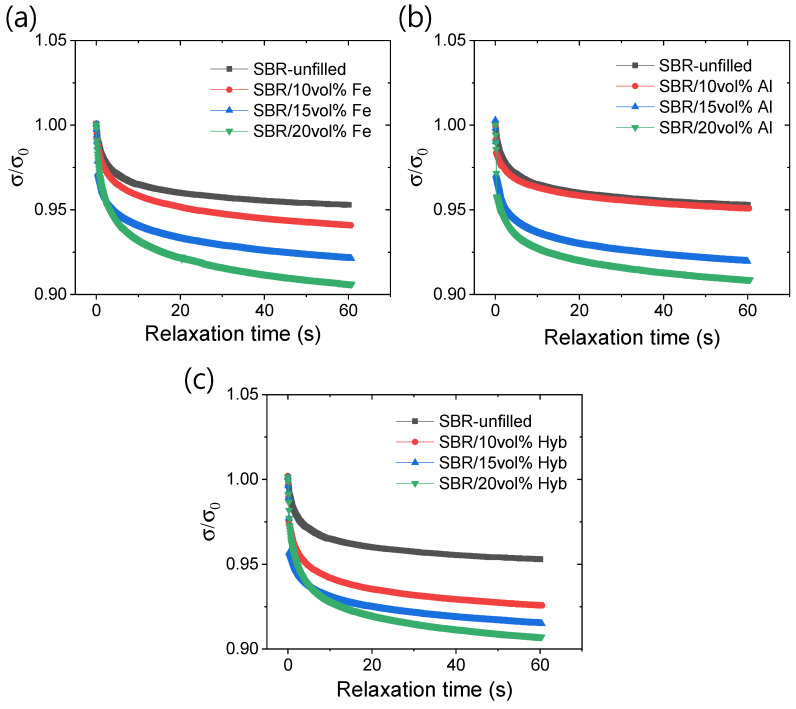
Stress relaxation behaviors; (**a**) iron-based composites, (**b**) aluminum-based composites, and (**c**) hybrid filler-based composites.

Figure 5a–c illustrates the stress relaxation behaviors of the rubber composites under a 30% applied strain. From the stress relaxation curves in Figure 5a–c, it is evident that relaxation rates increase with higher filler amounts. This phenomenon is likely due to the increased Young’s modulus and enhanced rigidity upon the addition of fillers. It is noticeable from Figure 5a–c that significant stress relaxation occurs within 10 s of the relaxation time. Iron-based composites exhibit prolonged relaxation times, indicating higher viscoelastic behavior compared to aluminum-based composites. The increased viscoelasticity in iron-based composites may be attributed to physical interactions between the π-electrons of benzene in SBR and the d-electrons in iron [27,32,33]. In contrast, aluminum-based composites, lacking such interactions, relax more quickly than their iron-containing counterparts.

### 3.2. Magneto-Mechanical and Electrical Properties

**Figure 6:**
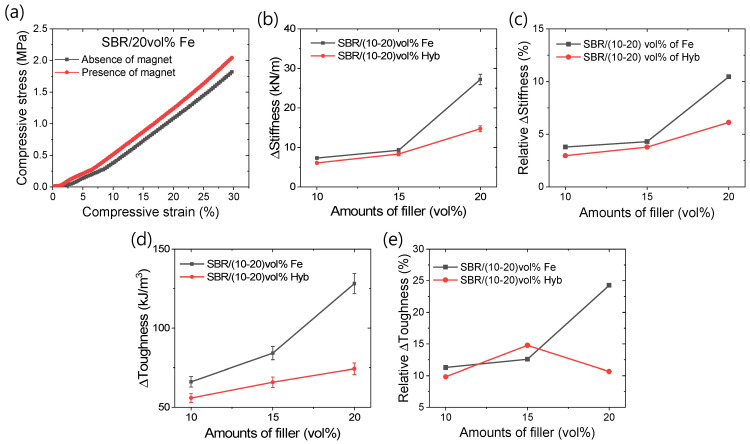
Magneto-mechanical properties; (**a**) typical stress–strain curves showing magnetic effect, (**b**) change in stiffness, (**c**) relative change in stiffness, (**d**) change in toughness, and (**e**) relative change in toughness.

Iron is a ferromagnetic material, imparting an anisotropic effect to iron-containing rubber composites when subjected to an external magnetic field [34,35,36,37]. This effect enhances the stiffness of magnetorheological elastomers, requiring more energy to deform or reducing the deformation amplitude. Consequently, they can effectively dampen significant forces and find utility as dampers in various mechanical systems [36,37]. Figure 6a–e present investigations into the anisotropic effects on different compressive mechanical properties. Figure 6a illustrates the typical compressive stress–strain curves in the presence and absence of magnetic fields, demonstrating the enhanced stiffness of magnetorheological elastomers and their potential in mechanical damping systems. Figure 6b,c depict changes in the stiffness and the relative changes induced by a permanent magnet in the composites with varying iron content. It is evident that the highest changes in stiffness and relative changes occur in composites with the highest iron content. For instance, the change in stiffness can exceed 25 kN/m for the 20 vol% iron-containing rubber composite with 1.2 T of magnetic field. Figure 6d,e illustrate changes in the toughness and relative toughness under magnetic field conditions. It shows that ΔToughness increases with the increasing iron content in the rubber composites. However, the relative toughness may decrease depending on the initial toughness of the composite. From Figure 6e, it can be observed that a composite with 20 vol% iron exhibits 25% higher relative toughness at 30% deformation. This suggests that applying a 1.2 T magnetic field can dampen 25% more energy due to the filler anisotropy. However, a higher damping efficiency is achieved at lower deformation levels, as the rubber composites become stiffer with increased deformation, leading to a reduced relative change in the stiffness values [28,38]. Significant changes in the stiffness and toughness values in the presence of a magnetic field, particularly at 20 vol% iron content, indicate enhanced filler percolation at this filler concentration.

**Figure 7:**
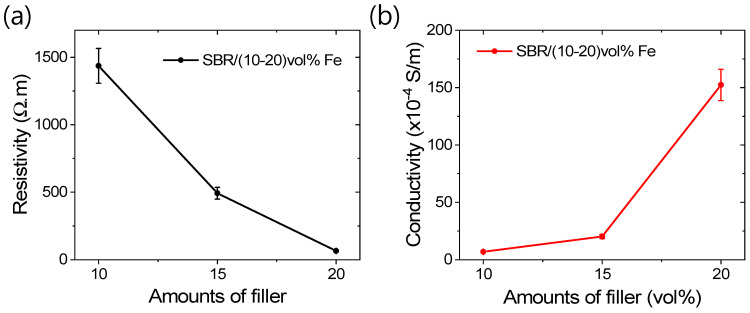
Electrical properties; (**a**) resistivity and (**b**) conductivity.

Although all the composites contain metal fillers, they do not exhibit significant electrical conductivity until the filler particles percolate to form conductive paths. It is noteworthy that iron, as a single filler system, demonstrates notable electrical conductivity, as shown and measured in Figure 7a,b. From Figure 7a,b, it is evident that significantly lower resistivity and higher electrical conductivity in the rubber composite can be achieved with a 20 vol% iron content. This level of iron content indicates that filler particles are sufficiently close to form effective conductive paths, thereby enhancing both the magnetic and electrical properties. The improved electrical conductivity and magnetic properties of these magnetic rubber composites render them ideal for a diverse range of applications in advanced mechanical and electronic devices.

### 3.3. Morphology of Rubber Composites

**Figure 8:**
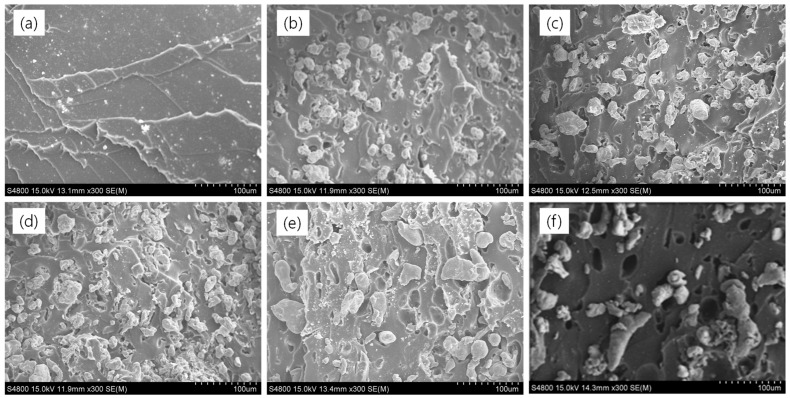
SEM images; (**a**) SBR-unfilled, (**b**) SBR/10vol% Fe, (**c**) SBR/15vol% Fe, (**d**) SBR/20vol% Fe, (**e**) SBR/20vol% Al, and (**f**) SBR/20vol% Hyb.

**Figure 9:**
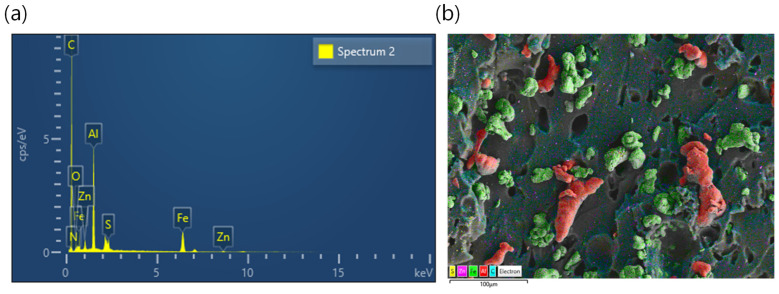
(**a**) Energy-dispersive X-ray spectroscopy of SBR/20vol% Hyb composite and (**b**) mapping of major elements in SBR/20vol% Hyb composite including iron (green) and aluminum (red) particles.

Figure 8a–f presents SEM images of both unfilled and filled rubber composites, revealing critical insights into their microstructures. The unfilled rubber composites (Figure 8a) appear smooth without significant features. In contrast, the filled rubber composites exhibit numerous vacuoles, indicating weak filler–rubber interactions. These composites are prone to tearing under tensile loading and show reduced tensile strength compared to the unfilled rubber. In iron-containing composites (Figure 8b–d), aggregated filler structures are evident, promoting electrical conductivity. This characteristic enhances their magneto-mechanical properties and results in a higher anisotropic effect due to improved filler particle contacts. Conversely, aluminum-based composites (Figure 8e) and hybrid filler systems (Figure 8f) demonstrate filler particles that are either separated or fail to form proper conductive channels, even at high filler concentrations. Consequently, these composites exhibit weaker filler–rubber interactions and inferior mechanical properties compared to iron-only composites. Moreover, iron-only composites show in-rubber structures that confirm their superior compressive modulus at higher filler contents. To further verify the filler distributions in the hybrid composite, energy-dispersive X-ray spectroscopy was conducted, as shown in Figure 9a,b. Figure 9a confirms the presence of elements such as C, N, O, S, Zn, Al, and Fe in the composites, originating from either curatives or added fillers. Elemental mapping of the major elements in Figure 9b indicates that while there is no effective filler networking for electrical conductivity, the composite exhibits magnetic properties due to the iron particles. Overall, these SEM observations underscore the significant impact of filler distribution and interactions on the mechanical and electrical properties of rubber composites, highlighting the advantages of iron-based composites in magneto-mechanical applications.

### 3.4. Swelling and Chemical Crosslink Densities

**Figure 10:**
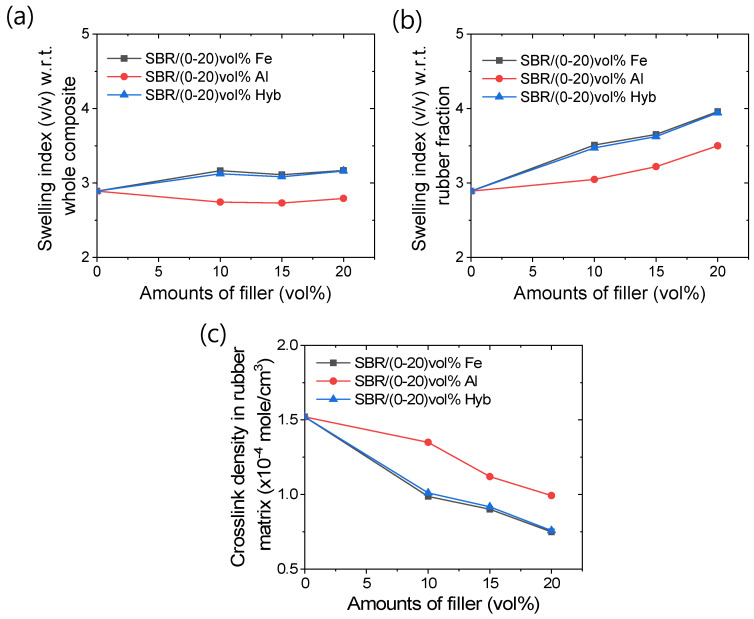
Swelling characteristics; (**a**) swelling index with respect to whole composites, (**b**) swelling index with respect to the rubber faction, (**c**) crosslink density in the rubber fraction.

**Figure 11:**
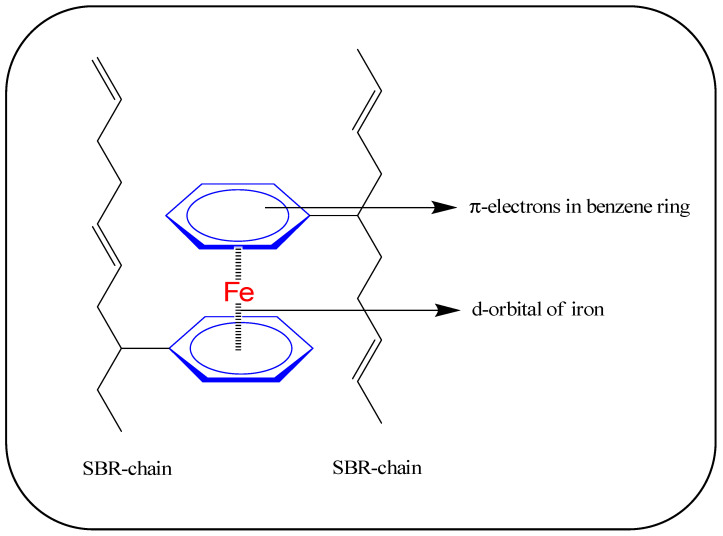
Possible interactions between π-electrons of the benzene ring in the SBR-chain and the d-orbital of iron.

Figure 10a–c illustrates the swelling behavior and crosslink density values of different rubber composites. In Figure 10a,b, the swelling index values are depicted for the entire composites and for the rubber matrix alone, respectively. From Figure 10a, there appear to be no significant differences in the swelling index values with increasing filler content in specific filler systems. However, Figure 10b shows that swelling index values increase with higher filler amounts. These results suggest that the fillers themselves do not significantly swell in toluene. Moreover, increasing the filler content can reduce the number of crosslinks in the rubber matrix, as shown in Figure 10c. Figure 10c demonstrates that metal composites generally exhibit reduced crosslink density compared to unfilled rubber, with aluminum-based composites showing better crosslink densities than other composites. Mechanical properties, such as modulus values of the composites, heavily depend on factors like network density in the rubber matrix and physical or chemical interactions between rubber and fillers. Reinforcement in these properties, particularly modulus values, primarily occurs through physical interactions such as filler–filler and filler–rubber interactions. From Figure 10c, it is also evident that iron particles have a more detrimental effect on crosslinks compared to aluminum particles. Anuar et al. [18] similarly found that zinc-based rubber composites exhibit lower mechanical properties and thermal stability compared to aluminum-based natural rubber composites, suggesting a higher destructive effect on crosslinks in the former. It is plausible that d-block elements like iron and copper exhibit higher reactivity with vulcanizing ingredients compared to p-block metals like aluminum, resulting in reduced crosslink density. Additionally, the antioxidant behavior or higher thermal conductivity of metals may inhibit vulcanization or decompose certain curatives before the formation of active sulfurating complexes [16,39,40]. Since vulcanization follows first-order kinetics with respect to the active sulfurating complex, a reduced amount of this complex may lead to fewer sulfur crosslinks in the rubber matrix [39,40]. Consequently, rubber–metal composites typically exhibit lower crosslink density values compared to unfilled rubber compounds. Despite iron-based composites having lower crosslink density than aluminum-based composites, their tensile toughness values are significantly better, suggesting stronger rubber–filler physical interactions, possibly through π-electron systems of rubber with d-electrons of iron [27] as illustrated in Figure 11.

## 4. Conclusions

Various metal powders, including iron, aluminum, and a 1:1 (*v*/*v*) hybrid, were studied as fillers in styrene–butadiene rubber (SBR). The mechanical properties of these composites indicate that all metal fillers can enhance the modulus values due to their physical interactions with the rubber. However, in many cases, metal fillers act as non-reinforcing fillers in SBR-based composites. This limitation is attributed to the micron size of the filler particles and their detrimental effects on chemical crosslink formation in the rubber matrix. Nevertheless, iron-based rubber composites exhibit exceptional magneto-mechanical properties and excellent electrical conductivity compared to other composites. At 20 vol% iron content, the composite exhibits a 25% relative change in toughness at 30% compression when exposed to a 1.2 T magnetic field, compared to the absence of the magnetic field. Additionally, this composite with 20 vol% iron content demonstrates electrical percolation, with an electrical conductivity of 1.5 × 10^−2^ S/m. It has been observed that iron, as a d-block element, has a more pronounced detrimental effect on crosslink formation than aluminum, which is a p-block element. The superior electrical and magnetic properties of iron-based rubber composites make them promising for applications in magnetorheology, sensors, electronics, and other advanced engineering systems. However, to address their poor mechanical properties, it is necessary to incorporate some amount of reinforcing filler such as carbon black or nano-silica to enhance overall performance.

## Figures and Tables

**Table 1:** Mix compositions of materials by actual weight taken in grams.

Formulation	Amount of Masterbatch Rubber (vol%)	Amount of Iron (vol%)	Amount of Aluminum (vol%)	Hybrid (1:1 *v*/*v*)	
				Amount of Iron (vol%)	Amount of Aluminum (vol%)
SBR-unfilled	23.5 (100) *	-	-	-	
SBR/10vol% Fe	21.15 (90)	19.68 (10)	-	-	
SBR/15vol% Fe	19.98 (85)	29.51 (15)	-	-	
SBR/20vol% Fe	18.8 (80)	39.35 (20)	-	-	
SBR/10vol% Al	21.15 (90)	-	6.75 (10)	-	
SBR/15vol% Al	19.98 (85)	-	10.13 (15)	-	
SBR/20vol% Al	18.8 (80)	-	13.5 (20)	-	
SBR/10vol% Hyb	21.15 (90)	-	-	9.84 (5)	3.38 (5)
SBR/15vol% Hyb	19.98 (85)	-	-	14.76 (7.5)	5.07 (7.5)
SBR/20vol% Hyb	18.8 (80)			19.68 (10)	6.75 (10)

* The values in parentheses indicate the volume fraction of each component in the compounded rubber.

## Data Availability

Data are contained within the article.
